# Hybridization and rapid differentiation after secondary contact between the native green anole (*Anolis carolinensis*) and the introduced green anole (*Anolis porcatus*)

**DOI:** 10.1002/ece3.5042

**Published:** 2019-03-26

**Authors:** Johanna E. Wegener, Jessica N. Pita‐Aquino, Jessica Atutubo, Adam Moreno, Jason J. Kolbe

**Affiliations:** ^1^ Department of Biological Sciences University of Rhode Island Kingston Rhode Island; ^2^ Department of Ecology and Evolutionary Biology Brown University Providence Rhode Island; ^3^ Present address: College of Veterinary Medicine Ohio State University Columbus Ohio

**Keywords:** approximate Bayesian computation, cytonuclear discordance, reproductive isolation

## Abstract

In allopatric species, reproductive isolation evolves through the accumulation of genetic incompatibilities. The degree of divergence required for complete reproductive isolation is highly variable across taxa, which makes the outcome of secondary contact between allopatric species unpredictable. Since before the Pliocene, two species of *Anolis* lizards, *Anolis carolinensis* and *Anolis porcatus*, have been allopatric, yet this period of independent evolution has not led to substantial species‐specific morphological differentiation, and therefore, they might not be reproductively isolated. In this study, we determined the genetic consequences of localized, secondary contact between the native green anole, *A. carolinensis*, and the introduced Cuban green anole, *A. porcatus*, in South Miami. Using 18 microsatellite markers, we found that the South Miami population formed a genetic cluster distinct from both parental species. Mitochondrial DNA revealed maternal *A. porcatus* ancestry for 35% of the individuals sampled from this population, indicating a high degree of cytonuclear discordance. Thus, hybridization with *A. porcatus*, not just population structure within *A. carolinensis*, may be responsible for the genetic distinctiveness of this population. Using tree‐based maximum‐likelihood analysis, we found support for a more recent, secondary introduction of *A. porcatus* to Florida. Evidence that ~33% of the nuclear DNA resulted from a secondary introduction supports the hybrid origin of the green anole population in South Miami. We used multiple lines of evidence and multiple genetic markers to reconstruct otherwise cryptic patterns of species introduction and hybridization. Genetic evidence for a lack of reproductive isolation, as well as morphological similarities between the two species, supports revising the taxonomy of *A. carolinensis *to include *A. porcatus *from western Cuba. Future studies should target the current geographic extent of introgression originating from the past injection of genetic material from Cuban green anoles and determine the consequences for the evolutionary trajectory of green anole populations in southern Florida.

## 1 INTRODUCTION

In allopatric species, reproductive isolation evolves through the accumulation of genetic incompatibilities in geographically separated lineages (Dobzhansky, 1937; Orr, 1995). As divergence time increases, negative epistatic interactions that reduce hybrid viability become more likely. However, the degree of divergence required for complete reproductive isolation is highly variable across taxa (Bolnick & Near, 2005; Martin et al., 2017; Stelkens, Young, & Seehausen, 2010; Wiens, Engstrom, & Chippindale, 2006), making the consequences of secondary contact between allopatric species unpredictable. When reproductive barriers are weak, secondary contact between previously isolated lineages (e.g., native and introduced species) can lead to hybridization (Prentis, Wilson, Dormontt, Richardson, & Lowe, 2008; Schierenbeck & Ellstrand, 2009), rapidly homogenize parental genotypes, erode species boundaries (Glotzbecker, Walters, & Blum, 2016; Hasselman et al., 2014; James & Abbott, 2005; Ward et al., 2012), and threaten the genetic integrity of native species (Brennan et al., 2015; Jiggins & Mallet, 2000). Because species introductions are often pulse‐like and localized as opposed to active hybrid zones, recombination and repeated backcrossing can result in complete admixture of parental genotypes and erase genetic signatures of hybridization within only few generations (Glotzbecker et al., 2016; Hasselman et al., 2014; Lombaert et al., 2011; Roy, Lucek, Walter, & Seehausen, 2015; Ward et al., 2012).

Empirical studies that document genetically cryptic hybridization patterns are rare (James & Abbott, 2005; Keller, Fields, Berardi, & Taylor, 2014; Kronforst, Young, Blume, Gilbert, & McMillan, 2006; Lavretsky, Engilis, Eadie, & Peters, 2015; Mims, Darrin Hulsey, Fitzpatrick, & Todd Streelman, 2010), and strong inferences often require sampling of reference populations of parental species as well as cytoplasmic and nuclear markers. Reference populations are necessary because repeated recombination and backcrossing can homogenize nuclear genotypes, which makes it challenging to distinguish hybrid populations from subpopulations of parental species (Della Croce, Poole, & Luikart, 2016). Non‐recombining cytoplasmic markers retain parental genotypes and thus can help to distinguish hybrid populations from population structure (Della Croce et al., 2016). A mismatch between nuclear genotypes and cytoplasmic haplotypes is often the first step to identifying hybrid populations (Toews & Brelsford, 2012). Subsequent population genetic analyses are then needed to distinguish between cytoplasmic introgression, past (including ancient) hybridization, and incomplete lineage sorting (Della Croce et al., 2016). Identifying past hybridization events between introduced and native species is particularly challenging since the geographic location of the source population is often unknown or includes multiple source locations, and pure native populations might be genetically swamped by introduced genotypes (Caracristi & Schlötterer, 2003; Della Croce et al., 2016; Kolbe et al., 2004, 2007; Kronforst et al., 2006). In this study, we aimed to reconstruct the invasion history of the Cuban green anole, *Anolis porcatus*, and determine the genetic consequences of localized, secondary contact with the native green anole, *Anolis carolinensis*, in southern Florida (USA). We use multilocus nuclear genotypes and mitochondrial haplotypes to distinguish between contemporary and past gene flow, allowing us to test whether secondary contact has eroded putative species boundaries or whether the two sister species are reproductively isolated and coexist as genetically distinct taxonomic units.

*Anolis porcatus* and *A. carolinensis* are allopatric species and have been geographically separated for an estimated 6–12 million years, since before the Pliocene (Campbell‐Staton et al., 2012; Manthey, Tollis, Lemmon, Moriarty Lemmon, & Boissinot, 2016; Tollis & Boissinot, 2014). *Anolis carolinensis* is nested within a clade of *A. porcatus* from western Cuba, making the latter species paraphyletic (Glor et al., 2004; Glor, Losos, & Larson, 2005). After the initial colonization of the Florida Peninsula, *A. carolinensis* has undergone substantial range expansion and differentiation resulting in five major mitochondrial clades. The current distribution ranges from southern Florida to North Carolina and west to Texas (Campbell‐Staton et al., 2012; Manthey et al., 2016; Tollis & Boissinot, 2014).

The introduction of *A. porcatus* in Florida was first suggested in the 1990s based on morphological characters (Meshaka, Clouse, Butterfield, & Hauge, 1997) and later confirmed genetically (Kolbe et al., 2007). Two individuals collected in Miami were genetically similar to *A. porcatus *in western Cuba, indicating the putative source population of the introduction (Kolbe et al., 2007). Since the 1940s, seven other non‐native anole species from various locations in Cuba and in the Caribbean have established in Miami, leading to admixture among genetically distinct source populations in several cases (Kolbe et al., 2007). Despite widespread intraspecific admixture, hybridization between recognized species is considered rare among anoles (Losos, 2009). A few cases are documented between closely related species, including *A. porcatus* × *Anolis allisoni* in central Cuba (Glor et al., 2004) and *Anolis pulchellus* × *Anolis krugi* in Puerto Rico (Jezkova, Leal, & Rodríguez‐Robles, 2013). Hybridization between *A. carolinensis* and *A. porcatus *has been suggested repeatedly, mainly because the two species have no species‐specific morphological characters despite considerable divergence time (Camposano, 2011; Kolbe et al., 2007; Tollis, 2013). Sufficient evidence for reproductive isolation or lack thereof has not been shown.

In this study, we examine whether *A. porcatus* and *A. carolinensis* are reproductively isolated species, and characterize the genetic consequences of secondary contact in South Miami. We used one mtDNA marker and 18 nuclear microsatellite loci to test whether hybridization has occurred between the two species. We distinguished between contemporary and historic gene flow and estimated the timing of the admixture event. Discordance between nuclear and cytoplasmic markers is characteristic of hybridization and commonly used to identify hybrid individuals (Toews & Brelsford, 2012). If the two species interbreed in South Miami, we expect a high frequency of individuals with mismatches between nuclear genotypes and mtDNA haplotypes. To distinguish between contemporary, ongoing gene flow (such as expected in an active hybrid zone) versus a limited, historic gene flow event (such as common in human‐mediated introductions), we used a genetic cluster analysis and a tree‐based analysis allowing for migration between previously separated lineages. In the case of contemporary gene flow, we expect genetic clusters that reflect the two parental lineages and an admixed cluster in which individuals carry nuclear DNA from both parental lineages. In the case of an historic gene flow event, genetic admixture is likely erased, leading to an independent genetic cluster for the hybrid population in which the genetic variation is the result of historic, but not ongoing gene flow.

## 2 MATERIALS AND METHODS

### 2.1 Sample collection

We sampled 32 *A. carolinensis* individuals from the J.W. Corbett Wildlife Management in southern Florida, which is ~135 km north of Miami, 92 green anole individuals from the putative hybrid population in South Miami, and 54 *A. porcatus* individuals from western Cuba (Supporting Information Table S1). Genomic DNA was extracted from tail tips and liver tissue using a modified ethanol precipitation protocol.

### 2.2 Molecular methods

We amplified a region of 343–571 bp of the mtDNA NADH dehydrogenase subunit two using primers from Campbell‐Staton et al. (2012) and two newly designed primers (Supporting Information Table S2). A 50 µl reaction contained 5.0 µl of 10× standard PCR buffer (New England Biolabs® Inc.), 3.0 µl of 10 mM dNTPs, 5.0 µl of 25 mM MgCl, 1.0 µl of 10 µM primer, 0.1 µl of 5 units/µl *Taq* polymerase (New England Biolabs® Inc.), and 4 µl of 50 ng/µl genomic DNA. Cycles started with initial denaturation at 94°C for 2 min, followed by 35 cycles of 94°C for 45 s, *T*_m_ for 45 s, 72°C for 1 min, and a final elongation step at 72°C for 10 min. PCR products were purified and sequenced at the Rhode Island Genomics and Sequencing Center.

We amplified 18 microsatellite markers using PCR with fluorescently labeled primers. We used seven newly designed primers (Supporting Information Table S1) and 11 previously published primers (Wordley, Slate, & Stapley, 2011). A 10 µl reaction contained 0.8 µl of 10× standard PCR buffer (New England Biolabs® Inc.), 0.8 µl of 10× BSA, 0.6 µl of 10 mM dNTPs, 1.50 µl of 25 mM MgCl, 0.24 µl of 10 µM primer, 0.08 µl of 5 units/µl *Taq* polymerase (New England Biolabs® Inc.), and 2 µl of 20 ng/µl genomic DNA. Cycles started with initial denaturation at 94°C for 2 min, followed by 35 cycles of 94°C for 45 s, *T*_m_ for 45 s, 72°C for 1 min, and a final elongation step at 72°C for 10 min. Samples were genotyped at the DNA Analysis Facility on Science Hill at Yale University. Markers for all samples were analyzed with the software GeneMapper® v4.1 and visually checked to ensure accurate peak calling.

### 2.3 Phylogenetic analysis and haplotype divergence

**Figure 1:**
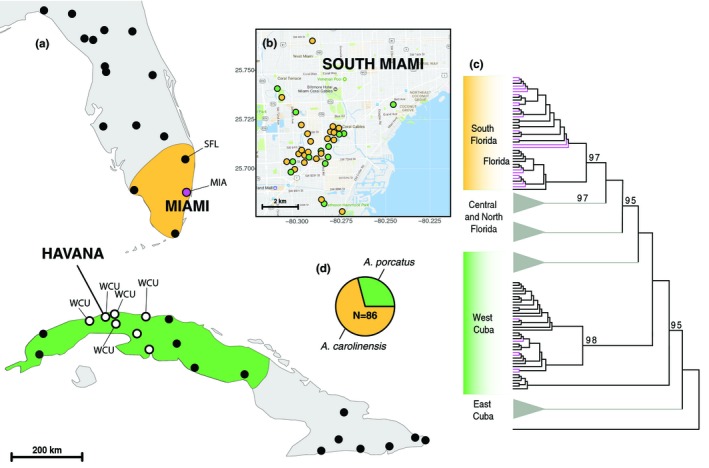
Sampling locations of mtDNA haplotypes and microsatellite data. (a) Black circles are sampling locations of mtDNA haplotypes. White circles indicate putative sources for introduced *Anolis porcatus*. Microsatellite data were sampled from five locations in western Cuba (WCU), from the putative hybrid population in South Miami (MIA) and ~135 km north of Miami (SFL). (b) Sampling sites in South Miami are colored by clade membership with yellow =* Anolis carolinensis*, green =* A. porcatus*. (c) Maximum‐likelihood phylogeny based on 571‐bp mtDNA haplotypes. Haplotypes from South Miami (magenta colored branches) are nested within both *A. porcatus* and *A. carolinensis*. Bootstrap values are shown above branches for values >95. Clades without haplotypes from South Miami were collapsed. The full phylogeny can be accessed in Supporting Information Figure S2. (d) Frequency of mtDNA haplotypes in South Miami, the total number of individuals sampled was *N* = 86

To determine the species identity of mtDNA haplotypes for individuals sampled in South Miami and the geographic origin of the introduction, we constructed a maximum‐likelihood phylogeny including samples from the geographic range of both species (Figure 1; Supporting Information Table S3). We combined previously published sequences with newly sampled individuals (see Supporting Information Table S3) resulting in 111 individuals of *A. porcatus* from eastern and western Cuba spanning the entire native range, 83 individuals of *A. carolinensis* sampled throughout Florida and 86 individuals from South Miami (Figure 1a). *Anolis loysiana* was used as outgroup taxon. Sequences were aligned and visually inspected for accuracy using the MUSCLE plugin in Geneious v7.1.9 (Kearse et al., 2012). We collapsed individual sequences into distinct haplotypes using DNAcollapser implemented in FaBox v1.41 (Villesen, 2007). To retain individuals with short mtDNA sequences, we generated two separate alignments. One alignment consisted of 571 bp for 200 individuals, resulting in 156 haplotypes. The second alignment was 343 bp long and included all 280 samples resulting in 182 haplotypes. We used RAxML v8.0 (Stamatakis, 2006) implemented in the CIPRES Science Gateway v3.3 (Miller, Pfeiffer, & Schwartz, 2011) to generate maximum‐likelihood phylogenies. Bootstrap values were obtained from 1,000 iterations using rapid bootstrapping.

We used pairwise sequence divergence to determine the degree of nucleotide divergence between native Cuban and introduced Florida *A. porcatus* haplotypes. We identified the genetically most similar individuals based on the fewest number of pairwise nucleotide differences. Pairwise sequence divergence was calculated as the number of nucleotide differences divided by the sequence length.

### 2.4 Population genetic statistics

In addition to one mtDNA locus, we genotyped 18 microsatellite loci for lizards sampled from the putative hybrid population in South Miami (MIA), five sampling locations of *A. porcatus* from western Cuba (WCU), and *A. carolinensis* from one sampling location 135 km north of Miami (SFL; Figure 1). We calculated deviations from Hardy–Weinberg equilibrium (HWE) and pairwise *F*_ST_ values in Genepop v1.2 (Rousset, 2008). Allelic richness and heterozygosity were calculated using the R package *Poppr *v2.2.0 (Kamvar, Brooks, & Grünwald, 2015; Kamvar, Tabima, & Grünwald, 2014).

### 2.5 Population structure and differentiation

First, we performed a Bayesian cluster analysis with STRUCTURE v2.3.4 (Rosenberg, 2004), using the admixture model and correlated allele frequencies. We allowed for gene flow among populations and modeled six different clustering scenarios, sequentially increasing the number of clusters from *K* = 1–6. We conducted 10 independent runs for each scenario with a burn‐in of 500,000 and 1,000,000 MCMC iterations. We used delta *K* to determine the most likely number of clusters following the Evanno method (Evanno, Regnaut, & Goudet, 2005) implemented in STRUCTURE HARVESTER v0.6.94 (Earl & Vonholdt, 2012). We combined the genotype proportions of each cluster (*q*‐matrix) from 10 independent runs with CLUMPP (Jakobsson & Rosenberg, 2007) and visualized the results with the R package ggplot2 v2.1.0 (Wickham, 2011). We repeated the Bayesian cluster analysis with population pairs (SFL–MIA and WCU–MIA) as well as WCU separately to identify potential population substructure. Model parameters were used as described above. Second, we used discriminant analysis of principal components (DAPC) to determine the degree of differentiation between clusters using the R package *adegenet* (Jombart, 2008). To characterize and find genetic clusters, DAPC uses a multivariate approach and PCA transformed allele frequencies. In contrast to the Bayesian clustering approach, DAPC does not rely on specific population model assumptions, such as HWE. The number of clusters (*K*) was sequentially increased starting with one cluster. The model fit for *K* clusters was determined with the Bayesian information criterion (BIC).

### 2.6 Maximum likelihood and ABC modeling of historic admixture

To detect historic gene flow, we used a tree‐based maximum‐likelihood approach with the program TreeMix (Pickrell & Pritchard, 2012). This approach uses allele frequencies to model relatedness among populations as a non‐bifurcating tree. Migration edges are added as additional branches to a bifurcating tree allowing for population ancestry from more than one parental population. Migration edges are added stepwise to the tree model until the covariance of the model best matches the covariance of the data. Residual matrices were used to determine the model fit. Positive residuals indicate greater genetic variation in the population than explained by the simple tree model suggesting admixture (Pickrell & Pritchard, 2012). The model assumes migration within a single generation. The fraction of alleles derived from migration is represented as weight of the migration edge.

To infer the timing of the admixture event, we used approximate Bayesian computation to model the demographic history of the three populations using DIY‐ABC v2.0 (Cornuet et al., 2014). A set of summary statistics was used to assess the fit between simulated datasets and empirical data. We used mean number of alleles, mean genetic diversity, pairwise *F*_ST_ values and the maximum‐likelihood coefficient of admixture *λ* (Choisy, Franck, & Cornuet, 2004). The demographic scenario simulates divergence between SFL and WCU and a more recent admixture event that gave rise to the MIA population. The prior for the divergence between SFL and WCU was set between (6,000,000–13,000,000) generations, based on previous divergence time estimates (Campbell‐Staton et al., 2012; Tollis & Boissinot, 2014). We set the prior for the effective population size as (100–10,000) using a uniform prior distribution. To estimate timing of the admixture event, we used a prior of (1–4,000) generations assuming one generation per year. We simulated 1,000,000 datasets and used the 1,000 datasets with the smallest Euclidean distance to the empirical data for parameter estimation.

## 3 RESULTS

### 3.1 Phylogenetic analysis and mtDNA haplotype divergence

The 571‐bp alignment resulted in a total of 155 haplotypes, 41 from southern Florida, 43 from central and northern Florida, 52 from western Cuba, and 19 from eastern Cuba. *Anolis porcatus* from western Cuba was sister to the monophyletic *A. carolinensis* (Supporting Information Figure S1). Since the 343‐bp alignment resulted in an overall similar tree topology (Supporting Information Figure S2), we focused on the 571‐bp alignment in the following analyses. Individual haplotypes and sampling locations for the 343‐bp alignment can be accessed in the Supplementary Material.

Our maximum‐likelihood phylogenetic analysis of mtDNA haplotypes revealed that individuals sampled in South Miami (*N* = 86) were not monophyletic. Six haplotypes representing thirty samples (35%) were nested within a well‐supported clade of *A. porcatus* from western Cuba, whereas twenty haplotypes representing 56 samples (65%) clustered with *A. carolinensis* from southern Florida (Figure 1c). Haplotypes from *A. carolinensis* and *A. porcatus* were codistributed across the study area in South Miami (Figure 1b).

Introduced *A. porcatus* haplotypes were nested in a well‐supported clade of *A. porcatus* from seven sampling locations near Havana in western Cuba (Figure 1a). Branches within the clade were not well supported (bootstrap <95), which limits our ability to identify a more specific source location(s) for the introduction. Average sequence divergence between introduced *A. porcatus* haplotypes and the genetically most similar ones sampled from Cuba ranged from 0.0% to 1.75% divergence (mean = 1.14% ± 0.68%; Table 1). One individual from South Miami (MIA640) shared the same haplotype with one individual from Havana (JJK2796).

**Table 1:** Introduced mtDNA haplotypes of *Anolis porcatus* sampled in South Miami and the genetically most similar haplotypes of *A. porcatus* from western Cuba

mtDNA haplotype			
South Miami	West Cuba	Sampling location West Cuba	% divergence
H102	H101	8 (Glor et al., 2004)	1.23
H103	H101	8 (Glor et al., 2004)	0.7
H105	H100	10, 11 (Glor et al., 2004)	1.4
H106	H122 & H126	Havana & 9 (Glor et al., 2004)	1.75
H107	H122	Havana	1.75
H123	H123	Havana	0

Note

Haplotypes are shown for mtDNA haplotype length of 571 bp.

### 3.2 Genetic diversity and differentiation using nuclear microsatellite loci

Genetic structure and diversity were assessed for populations from WCU, SFL, and MIA using nuclear microsatellite markers. Three microsatellite loci (Ac2, F06, g01) deviated significantly (*p* < 0.05) from HWE. Excluding those loci from the analysis did not affect the results and we thus included them in subsequent analyses. Allelic richness was similar across populations (mean *A*_R_ = 10.62 ± 0.55; Table 2). Observed heterozygosity was lower than expected heterozygosity in all populations (mean *H*_o_ = 0.70 ± 0.03; mean *H*_e_ = 0.81 ± 0.03). *F*_ST_ values showed similar degrees of differentiation between populations (mean pairwise *F*_ST_ = 0.08 ± 0.01, Table 2). Individual allele frequencies of all markers are shown in Supporting Information Figure S2.

**Table 2:** Microsatellite summary statistics

Population	*N*	*A*_R_	*H*_o_	*H*_e_	*M*‐ratio	*F*_ST_—SFL	*F*_ST_—MIA
SFL	32	10.22	0.68	0.79	0.81		
MIA	92	10.38	0.68	0.81	0.85	0.09	
WCU	54	11.24	0.73	0.85	0.83	0.08	0.07

Notes

*A*_R_: allelic richness; *H*_e_: expected heterozygosity and pairwise *F*_ST_; *H*_o_: observed heterozygosity; *N*: number of individuals.

SFL = individuals from J.W. Corbett Wildlife Management in southern Florida; MIA = South Miami, FL; and WCU = western Cuba.

### 3.3 Population structure and differentiation

**Figure 2:**
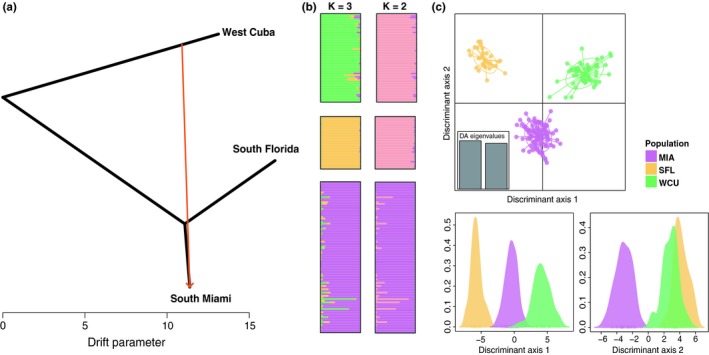
Historic gene flow and differentiation of the hybrid population in South Miami. (a) Tree‐based ancestry model with migration edge (red arrow) indicating gene flow between western Cuba (WCU) and South Miami (MIA) accounting for 33% of nuclear genetic variation in the MIA population. (b) Genetic clusters from the Bayesian cluster analysis for *K* = 2 and *K* = 3, the latter being the most likely number of clusters. (c) Discriminant analysis of principal components analysis with *K* = 3 clusters, PC axis 1 accounted for 51% of variation and PC axis 2 for 49%. Bottom figures show the density of each cluster for PC axis 1 (left) and PC axis 2 (right)

The Bayesian cluster analysis using STRUCTURE recovered three distinct genetic clusters (Figure 2b,c; model comparison Supporting Information Figure S4). Individual genotypes were correctly reassigned to their sampling locations and had genotype proportions >90% consistent with their own cluster. The MIA population shared a larger proportion of genotypes with WCU than with SFL, but this accounted for <5% of ancestry with the average genotype proportion assigned to WCU and SFL being 0.03 ± 0.05 and 0.01 ± 0.01, respectively. Three MIA individuals shared genotype proportions >20% with WCU (MIA647 *q* = 0.21 MIA719; *q* = 0.86 and MIA725 *q* = 0.70).

When the number of clusters was set to *K* = 2 in the STRUCTURE analysis, the MIA population remained a distinct cluster while SFL and WCU formed a single genetic cluster (Figure 2b). Analysis of separate population pairs recovered all three populations and did not suggest population substructure (Supporting Information Figure S6). Similarly, genotypes from five sampling locations in WCU showed no evidence for population structure when analyzed separately (Supporting Information Figure S7).

Consistent with the STRUCTURE results, the DAPC analysis revealed three distinct genetic clusters. All 178 individuals grouped according to the three sampling locations WCU, SFL, and MIA except for one individual that was sampled in WCU but assigned to the MIA cluster (posterior probability = 0.83). Similar distances between cluster centroids indicated equal degrees of genetic differentiation among the populations (Figure 2c; PCA for within‐cluster variation is shown in Supporting Information Figure S5). The MIA cluster was intermediate between SFL and WCU on the first PC axis. On the second PC axis, MIA was distinct with respect to SFL and WCU, which had similar values.

### 3.4 Maximum likelihood and ABC modeling of historic admixture

Tree‐based maximum‐likelihood analysis of microsatellite markers supported one migration event between WCU and MIA (Figure 2a). The weighted migration edge suggested that ~33% of the nuclear genetic ancestry in the MIA population is derived from WCU. Including the migration edge significantly improved the fit of the model as compared to a strictly bifurcating tree model (*p* < 0.001, Supporting Information Table S4). The migration model explained 99% of the total variance in the data, whereas the strictly bifurcating tree model accounted for only 80% (Supporting Information Figure S8).

**Figure 3:**
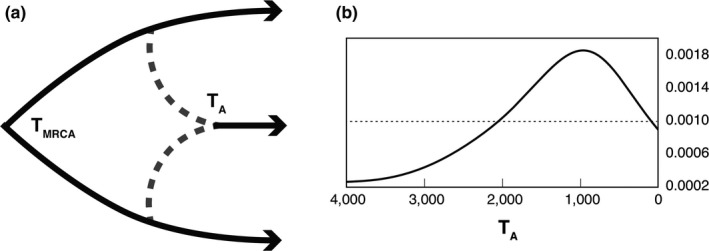
(a) Demographic ABC model and time of admixture between *Anolis carolinensis* and *Anolis porcatus *in South Miami. (b) Solid line shows the posterior distribution of *T*_A _in units of generations and the uniform prior distribution as dotted line. Median time of the admixture event was 887 with 95% CI 245–2,670

Time estimates from the ABC analysis suggest that the admixture event between MIA and WCU occurred within the last 245–2,670 generations (median *T*_A_ = 887, mode *T*_A_ = 554, 95% CI 245–2,670; Table 3, Figure 3). The median rate of admixture was *R*_A_ = 0.24 (95% CI 0.14–0.35), which is similar to the maximum‐likelihood coefficient of admixture *λ* = 0.31 obtained from the summary statistics (Supporting Information Table S5). Estimates for the remaining parameters used in the model are shown in Supporting Information Table S5. Summary statistics generated from the posterior probability distribution show similar values compared to the observed data and were largely nonsignificant, suggesting that modeled parameters provide a good fit for the data (Supporting Information Table S5). Performance measures for parameter estimates were consistently low, indicating accurate estimates (Supporting Information Table S6).

**Table 3:** Posterior parameter estimates from the ABC demographic scenario

Parameter	Median (95% CI)
*N*_SFL_	4,980 (2,130–9,030)
*N*_MIA_	4,410 (2,230–7,270)
*N*_WCU_	8,570 (5,670–9,920)
*T*_A_	887 (245–2,670)
*R*_A_	0.24 (0.14–0.35)
*T*_MRCA_	1.03* 10^6^ (1.28 × 10^6^–1.29 × 10^7^)

Note

*N*: effective population size; *R*_A_: admixture rate; *T*_A_: time of the admixture event in units of generations; *T*_MRCA_: time of the split between SFL and WCU.

## 4 DISCUSSION

In an effort to characterize the genetic consequences of secondary contact between the native *A. carolinensis* and the closely related introduced *A. porcatus*, our data show evidence for past hybridization followed by differentiation of the hybrid population. We found discordance between nuclear microsatellite markers and mtDNA haplotypes in the South Miami population, which is indicative of hybridization (Hailer et al., 2012; Miller et al., 2012; Roy et al., 2015). Thirty‐five per cent of mitochondrial haplotypes in the South Miami population are derived from *A. porcatus* from western Cuba and 65% from the native *A. carolinensis* in southern Florida, verifying the introduction of *A. porcatus* from Cuba and producing secondary contact with native *A. carolinensis* in southern Florida. Genetic cluster analyses of nuclear markers show that the South Miami population is homogeneous and genetically distinct from populations of both parental species, which is characteristic of hybrid ancestry rather than ongoing hybridization (James & Abbott, 2005; Keller et al., 2014; Kronforst et al., 2006; Lavretsky et al., 2015; Mims et al., 2010). Tree‐based maximum‐likelihood analysis confirms that ~33% of the nuclear genetic ancestry is derived from western Cuba via secondary gene flow. This proportion of nuclear ancestry (~33%) is strikingly similar to the proportion of *A. porcatus* mtDNA haplotypes in South Miami (35%). Thus, reproductive barriers between *A. porcatus* and *A. carolinensis* appear weak or absent despite divergence in allopatry since before the Pliocene. Thus, secondary contact after species introduction has led to hybridization and formed a genetically distinct green anole population of hybrid origin.

Time estimates from ABC analyses suggest that hybridization occurred between 245 and 2,670 generations ago with a skewed distribution toward the present (Figure 3), suggesting relatively recent introduction and rapid differentiation of the hybrid population. Surprisingly, the differentiation of the hybrid population in South Miami from both parental species is similar in magnitude to the differentiation between the parental species, *A. porcatus* and *A. carolinensis*. Potential factors facilitating differentiation include reduced gene flow with populations of the parental species (Hasselman et al., 2014; James & Abbott, 2005; Roy et al., 2015; Schumer, Cui, Powell, Rosenthal, & Andolfatto, 2016), assortative mating of hybrid individuals (Mavárez et al., 2006), increased hybrid fitness (e.g., heterosis; Schwarz, Matta, Shakir‐Botteri, & McPheron, 2005), and genome incompatibility (Schumer, Cui, Rosenthal, & Andolfatto, 2015). Certainly, the situation favors the potential for asymmetric gene flow biased against the geographically isolated *A. porcatus* from Cuba. Whether mainly adaptive or neutral evolutionary processes are involved in driving differentiation of the hybrid population and to what extent ongoing introgression exists in locations where hybrid and pure individuals overlap remain to be determined in future studies. However, preexisting population structure might have contributed to differentiation of the South Miami population in addition to hybridization.

This study demonstrates that multiple lines of evidence and multiple genetic markers are necessary to reconstruct cryptic patterns of species introduction and hybridization. Solely based on nuclear markers, the South Miami (MIA) population, which formed a distinct genetic cluster, would have been indistinguishable from within‐species population structure. However, addition of a non‐recombining mtDNA marker revealed maternal ancestry from both *A. porcatus *and *A. carolinensis*. Using tree‐based maximum‐likelihood analysis, we were able to distinguish between a secondary, more recent introduction of *A. porcatus* to Florida from cytoplasmic introgression and incomplete lineage sorting (Della Croce et al., 2016). Evidence that ~33% of the nuclear DNA resulted from a secondary introduction rather than independent evolution supports the hybrid origin of the MIA population and makes incomplete lineage sorting a less plausible explanation. Multiple analyses were needed in addition to nuclear and cytoplasmic markers to reconstruct the complex migration and gene flow patterns of *A. porcatus* and *A. carolinensis* in southern Florida.

Our study provides genetic evidence that the formerly independent lineages *A. carolinensis* from southern Florida and *A. porcatus* from western Cuba are not reproductively isolated and interbreed successfully after secondary contact, leading to a fusion of the previously distinct lineages. The species status of *A. porcatus* and *A. carolinensis* has changed repeatedly over the last decades based on morphological traits (Gray, 1840; Powell, 1992; Voigt, 1831). *Anolis porcatus* was considered a subspecies of *A. carolinensis* (Gray, 1845) until described as a distinct taxonomic unit (Powell, 1992). However, a thorough evaluation of morphological differences between the species concluded that morphological characters are inadequate for species delimitation (Camposano, 2011). Genetic evaluation of the *A. carolinensis* species complex revealed paraphyly of *A. porcatus*, dividing this species into eastern and western clades in Cuba, with the western clade being sister to *A. carolinensis* (Glor et al., 2004, 2005). Our study provides a genetic perspective on species boundaries between *A. carolinensis* and *A. porcatus*. According to the biological species concept, populations of distinct species are incapable of effectively interbreeding with one another (Mayr, 1982), which is inconsistent with the findings of our study. Thus, genetic evidence for successful hybridization, as well as morphological similarities between the two species (Camposano, 2011), supports revising the taxonomy of the clade of *A. porcatus *from western Cuba, which should be subsumed into the earlier named *A. carolinensis*.

Several *Anolis* species have been introduced to Florida and some from multiple native‐range source populations in Cuba (Kolbe et al., 2007). In agreement with previously collected *A. porcatus* haplotypes from Miami (Kolbe et al., 2007), our phylogenetic analysis identified sampling sites located near Havana in western Cuba as potential source of the introduction. Haplotypes from the western Cuba locations sampled in our study did not show evidence of geographic structure (Figure 1a), which is consistent with a single western Cuban population based on microsatellite data (Supporting Information Figure S6). Thus, the source locations likely resemble a single panmictic population. However, a more comprehensive sampling approach of western Cuban populations is needed to clarify whether population structure exists and whether the introduction of *A. porcatus* involves a single or multiple independent introductions.

## 5 CONCLUSION

The aim of this study was to characterize the genetic consequences of secondary contact between *A. porcatus* and *A. carolinensis* and to test whether weak or absent reproductive barriers have led to hybridization and erosion of putative species boundaries between green anoles in South Miami. Mismatch between cytoplasmic and nuclear DNA as well as genetic evidence for past gene flow supports that *A. porcatus *and *A. carolinensis *are not reproductively isolated and that secondary contact has led to hybridization and fusion of formerly independent lineages. Therefore, the western Cuban lineage of *A. porcatus *should be subsumed taxonomically into *A. carolinensis* given its priority (Gray, 1840; Powell, 1992; Voigt, 1831). A major finding was that a temporally restricted hybridization event resulted in strong differentiation between the hybrid population and populations of the two parental lineages with no evidence of ongoing gene flow. Only by using a combination of nuclear and non‐recombining cytoplasmic markers and analyses that distinguish between past and current gene flow, were we able to reconstruct the complex and otherwise cryptic migration patterns. Future studies should target the current geographic extent of introgression originating from the past injection of genetic material from Cuban green anoles and determine the consequences for the evolutionary trajectory of green anole populations in southern Florida.

## CONFLICT OF INTEREST

None declared.

## AUTHOR CONTRIBUTION

Johanna Wegener designed the project, collected specimens in the field, contributed substantially to data collection in the laboratory and supervision of undergraduate work, conducted the data analysis, data interpretation, manuscript writing, and revisions. Jessica N. Pita Aquino contributed substantially to data collection in the lab, interpretation of the data, and manuscript revisions. Jessica Atutubo and Adam Moreno contributed substantially by collecting data in the laboratory, interpreting data, and revising the manuscript. Dr. Jason J. Kolbe made substantial contributions by designing the project, by interpreting data analyses and results, and by playing a critical role throughout the manuscript revision process.

## Data Availability

The authors confirm that data supporting the findings of this study will be made available on GenBank and on GitHub (https://github.com/johannawegener/Wegener-et-al-2019). The authors agree with the policy on data archiving by the journal Ecology and Evolution.
