# Supplementary material for: Hybridization and rapid differentiation after secondary contact between the native green anole (Anolis carolinensis) and the introduced green anole (Anolis porcatus)
Source: Ecol Evol. 2019 Mar 26;9(7):4138–48. doi: 10.1002/ece3.5042 (PMC6468060; doi:10.1002/ece3.5042)
Supplement: Supplementary file 1 [file ECE3-9-4138-s001.docx]

**Supplementary Material**

**Table S1**. Sample ID, mtDNA haplotype, sampling location and mtDNA clade membership of samples included in the phylogenetic analysis. Haplotypes were generated from two alignments S = 343 bp, H = 571 bp.

| **Accession number** | **Sample ID** | **mtDNA Haplotype** | **Sampling location** | **Latitude** | **Longitude** | **mtDNA clade** |
| --- | --- | --- | --- | --- | --- | --- |
| MK509116 | JJK2782 | S164 | Varadero, Cuba | 23.19039 | -81.16894 | *A. porcatus* |
| MK509077 | JJK2784 | S105 | Varadero, Cuba | 23.19039 | -81.16894 | *A. porcatus* |
| MK509110 | JJK2786 | S147 | Varadero, Cuba | 23.19039 | -81.16894 | *A. porcatus* |
| MK509076 | JJK2787 | S104 | Varadero, Cuba | 23.19039 | -81.16894 | *A. porcatus* |
| MK509095 | JJK2788 | S123 | Varadero, Cuba | 23.19039 | -81.16894 | *A. porcatus* |
| MK509078 | JJK2789 | S105 | Varadero, Cuba | 23.19039 | -81.16894 | *A. porcatus* |
| MK509115 | JJK2790 | S163 | Varadero, Cuba | 23.19039 | -81.16894 | *A. porcatus* |
| MK509160 | JJK2793 | S142/H118 | Havana, Cuba | 23.08706 | -82.36572 | *A. porcatus* |
| MK509161 | JJK2794 | S144/H119 | Havana, Cuba | 23.08706 | -82.36572 | *A. porcatus* |
| MK509167 | JJK2795 | S148 | Havana, Cuba | 23.08706 | -82.36572 | *A. porcatus* |
| MK509164 | JJK2796 | S148/H122 | Havana, Cuba | 23.08706 | -82.36572 | *A. porcatus* |
| MK509158 | JJK2797 | S131/H122 | Havana, Cuba | 23.08706 | -82.36572 | *A. porcatus* |
| MK509159 | JJK2800 | S131/H113 | Havana, Cuba | 23.08706 | -82.36572 | *A. porcatus* |
| MK509165 | JJK2825 | S149/H123 | Havana, Cuba | 23.08706 | -82.36572 | *A. porcatus* |
| MK509107 | JJK2826 | S139 | Havana, Cuba | 23.08706 | -82.36572 | *A. porcatus* |
| MK509098 | JJK2827 | S126 | Havana, Cuba | 23.11684 | -82.38881 | *A. porcatus* |
| MK509109 | JJK2828 | S143 | Havana, Cuba | 23.11684 | -82.38881 | *A. porcatus* |
| MK509156 | JJK2829 | S128/H110 | Havana, Cuba | 23.11684 | -82.38881 | *A. porcatus* |
| MK509100 | JJK2832 | S129 | Havana, Cuba | 23.11684 | -82.38881 | *A. porcatus* |
| MK509162 | JJK2833 | S145/H120 | Havana, Cuba | 23.11684 | -82.38881 | *A. porcatus* |
| MK509163 | JJK2834 | S146/H121 | Havana, Cuba | 23.11684 | -82.38881 | *A. porcatus* |
| MK509157 | JJK2835 | S130/H111 | Havana, Cuba | 23.11684 | -82.38881 | *A. porcatus* |
| MK509074 | JJK2859 | S99 | Havana, Cuba | 23.11684 | -82.38881 | *A. porcatus* |
| MK509092 | JJK2984 | S121 | Mariel, Cuba | 22.98575 | -82.75347 | *A. porcatus* |
| MK509102 | JJK2985 | S132 | Mariel, Cuba | 22.98575 | -82.75347 | *A. porcatus* |
| MK509094 | JJK2986 | S123 | Mariel, Cuba | 22.98575 | -82.75347 | *A. porcatus* |
| MK509075 | JJK2989 | S100 | Mariel, Cuba | 22.98575 | -82.75347 | *A. porcatus* |
| MK509112 | JJK2991 | S151 | Mariel, Cuba | 22.98575 | -82.75347 | *A. porcatus* |
| MK509093 | JJK2992 | S122 | Mariel, Cuba | 22.98575 | -82.75347 | *A. porcatus* |
| MK509085 | JJK3003 | S116 | Mariel, Cuba | 22.98575 | -82.75347 | *A. porcatus* |
| MK509097 | JJK3026 | S125 | San Jose de las Lajas, Cuba | 22.96503 | -82.16134 | *A. porcatus* |
| MK509106 | JJK3027 | S137 | San Jose de las Lajas, Cuba | 22.96503 | -82.16134 | *A. porcatus* |
| MK509104 | JJK3028 | S135 | San Jose de las Lajas, Cuba | 22.96503 | -82.16134 | *A. porcatus* |
| MK509096 | JJK3029 | S124 | San Jose de las Lajas, Cuba | 22.96503 | -82.16134 | *A. porcatus* |
| MK509103 | JJK3030 | S134 | San Jose de las Lajas, Cuba | 22.96503 | -82.16134 | *A. porcatus* |
| MK509083 | JJK3031 | S113 | San Jose de las Lajas, Cuba | 22.96503 | -82.16134 | *A. porcatus* |
| MK509113 | JJK3032 | S152 | San Jose de las Lajas, Cuba | 22.96503 | -82.16134 | *A. porcatus* |
| MK509105 | JJK3066 | S136 | Guanabo, Cuba | 23.15459 | -82.10105 | *A. porcatus* |
| MK509114 | JJK3067 | S152 | Guanabo, Cuba | 23.15459 | -82.10105 | *A. porcatus* |
| MK509101 | JJK3068 | S130 | Guanabo, Cuba | 23.15459 | -82.10105 | *A. porcatus* |
| MK509111 | JJK3069 | S150 | Guanabo, Cuba | 23.15459 | -82.10105 | *A. porcatus* |
| MK509108 | JJK3070 | S140 | Guanabo, Cuba | 23.15459 | -82.10105 | *A. porcatus* |
| MK509099 | JJK3071 | S127 | Guanabo, Cuba | 23.15459 | -82.10105 | *A. porcatus* |
| MK509135 | MIA636 | S63/H56 | South Miami, FL | 25.703825 | -80.284162 | *A. carolinensis* |
| MK509087 | MIA637 | S118 | South Miami, FL | 25.703825 | -80.284162 | *A. porcatus* |
| MK509166 | MIA640 | S194/H123 | South Miami, FL | 25.732598 | -80.245682 | *A. porcatus* |
| MK509090 | MIA641 | S118 | South Miami, FL | 25.732598 | -80.245682 | *A. porcatus* |
| MK509088 | MIA642 | S118 | South Miami, FL | 25.705713 | -80.293224 | *A. porcatus* |
| MK509086 | MIA643 | S117 | South Miami, FL | 25.705713 | -80.293224 | *A. porcatus* |
| MK509141 | MIA644 | H103 | South Miami, FL | 25.705892 | -80.295908 | *A. porcatus* |
| MK509082 | MIA645 | S111 | South Miami, FL | 25.705892 | -80.295908 | *A. porcatus* |
| MK509118 | MIA646 | S29/H31 | South Miami, FL | 25.705892 | -80.295908 | *A. carolinensis* |
| MK509123 | MIA647 | S37/H38 | South Miami, FL | 25.705892 | -80.295908 | *A. carolinensis* |
| MK509155 | MIA648 | S118/H107 | South Miami, FL | 25.705892 | -80.295908 | *A. porcatus* |
| MK509130 | MIA649 | S50/H49 | South Miami, FL | 25.705892 | -80.295908 | *A. carolinensis* |
| MK509154 | MIA652 | S118/H107 | South Miami, FL | 25.705892 | -80.295908 | *A. porcatus* |
| MK509117 | MIA653 | S28/H30 | South Miami, FL | 25.707552 | -80.29912 | *A. carolinensis* |
| MK509051 | MIA654 | S52 | South Miami, FL | 25.707552 | -80.29912 | *A. carolinensis* |
| MK509138 | MIA655 | S78/H67 | South Miami, FL | 25.707552 | -80.29912 | *A. carolinensis* |
| MK509142 | MIA656 | S111/H103 | South Miami, FL | 25.707552 | -80.29912 | *A. porcatus* |
| MK509146 | MIA657 | S118/H107 | South Miami, FL | 25.707552 | -80.29912 | *A. porcatus* |
| MK509121 | MIA659 | S36/H36 | South Miami, FL | 25.706803 | -80.295817 | *A. carolinensis* |
| MK509153 | MIA660 | S118/H107 | South Miami, FL | 25.705713 | -80.293224 | *A. porcatus* |
| MK509126 | MIA661 | S39/H41 | South Miami, FL | 25.705713 | -80.293224 | *A. carolinensis* |
| MK509062 | MIA662 | S61 | South Miami, FL | 25.705713 | -80.293224 | *A. carolinensis* |
| MK509152 | MIA664 | S118/H107 | South Miami, FL | 25.705713 | -80.293224 | *A. porcatus* |
| MK509122 | MIA665 | S36/H36 | South Miami, FL | 25.705077 | -80.289196 | *A. carolinensis* |
| MK509054 | MIA667 | S50 | South Miami, FL | 25.699681 | -80.301318 | *A. carolinensis* |
| MK509064 | MIA668 | S62 | South Miami, FL | 25.703554 | -80.303461 | *A. carolinensis* |
| MK509079 | MIA670 | S111 | South Miami, FL | 25.703554 | -80.303461 | *A. porcatus* |
| MK509063 | MIA671 | S62 | South Miami, FL | 25.703554 | -80.303461 | *A. carolinensis* |
| MK509127 | MIA672 | S45/H46 | South Miami, FL | 25.703554 | -80.303461 | *A. carolinensis* |
| MK509068 | MIA673 | S68 | South Miami, FL | 25.703554 | -80.303461 | *A. carolinensis* |
| MK509081 | MIA674 | S111 | South Miami, FL | 25.701835 | -80.30337 | *A. porcatus* |
| MK509124 | MIA675 | S37/H39 | South Miami, FL | 25.701835 | -80.30337 | *A. carolinensis* |
| MK509050 | MIA676 | S51 | South Miami, FL | 25.703554 | -80.303461 | *A. carolinensis* |
| MK509047 | MIA677 | S48 | South Miami, FL | 25.713857 | -80.29235 | *A. carolinensis* |
| MK509169 | MIA680 | S53 | South Miami, FL | 25.7109 | -80.284038 | *A. carolinensis* |
| MK509080 | MIA681 | S111 | South Miami, FL | 25.7109 | -80.284038 | *A. porcatus* |
| MK509058 | MIA682 | S58 | South Miami, FL | 25.7109 | -80.284038 | *A. carolinensis* |
| MK509091 | MIA683 | S120 | South Miami, FL | 25.715213 | -80.283312 | *A. porcatus* |
| MK509049 | MIA684 | S49 | South Miami, FL | 25.764939 | -80.291341 | *A. carolinensis* |
| MK509143 | MIA685 | S111/H103 | South Miami, FL | 25.71784 | -80.273581 | *A. porcatus* |
| MK509070 | MIA686 | S71 | South Miami, FL | 25.71784 | -80.273581 | *A. carolinensis* |
| MK509120 | MIA688 | S34/H35 | South Miami, FL | 25.71784 | -80.273581 | *A. carolinensis* |
| MK509072 | MIA689 | S79 | South Miami, FL | 25.71784 | -80.273581 | *A. carolinensis* |
| MK509043 | MIA690 | S30 | South Miami, FL | 25.71533 | -80.280858 | *A. carolinensis* |
| MK509065 | MIA692 | S62 | South Miami, FL | 25.71533 | -80.280858 | *A. carolinensis* |
| MK509150 | MIA694 | S118/H107 | South Miami, FL | 25.71533 | -80.280858 | *A. porcatus* |
| MK509061 | MIA695 | S60 | South Miami, FL | 25.71533 | -80.280858 | *A. carolinensis* |
| MK509052 | MIA696 | S53 | South Miami, FL | 25.71533 | -80.280858 | *A. carolinensis* |
| MK509066 | MIA697 | S66 | South Miami, FL | 25.715779 | -80.281151 | *A. carolinensis* |
| MK509148 | MIA699 | S118/H107 | South Miami, FL | 25.715779 | -80.281151 | *A. porcatus* |
| MK509053 | MIA700 | S45 | South Miami, FL | 25.715779 | -80.281151 | *A. carolinensis* |
| MK509149 | MIA701 | S118/H107 | South Miami, FL | 25.715779 | -80.281151 | *A. porcatus* |
| MK509060 | MIA702 | S59 | South Miami, FL | 25.718418 | -80.279209 | *A. carolinensis* |
| MK509089 | MIA703 | S118 | South Miami, FL | 25.717406 | -80.276231 | *A. porcatus* |
| MK509151 | MIA705 | S118/H107 | South Miami, FL | 25.717406 | -80.276231 | *A. porcatus* |
| MK509133 | MIA709 | S50/H53 | South Miami, FL | 25.703825 | -80.284162 | *A. carolinensis* |
| MK509132 | MIA710 | S50/H53 | South Miami, FL | 25.703825 | -80.284162 | *A. carolinensis* |
| MK509128 | MIA712 | S46/H47 | South Miami, FL | 25.706499 | -80.2856 | *A. carolinensis* |
| MK509147 | MIA713 | S118/H107 | South Miami, FL | 25.706499 | -80.2856 | *A. porcatus* |
| MK509129 | MIA714 | S47/H48 | South Miami, FL | 25.706499 | -80.2856 | *A. carolinensis* |
| MK509136 | MIA715 | S65/H58 | South Miami, FL | 25.706499 | -80.2856 | *A. carolinensis* |
| MK509131 | MIA716 | S50/H53 | South Miami, FL | 25.706275 | -80.285502 | *A. carolinensis* |
| MK509139 | MIA717 | S78/H69 | South Miami, FL | 25.706275 | -80.285502 | *A. carolinensis* |
| MK509125 | MIA718 | S38/H40 | South Miami, FL | 25.706275 | -80.285502 | *A. carolinensis* |
| MK509137 | MIA719 | S68/H59 | South Miami, FL | 25.706275 | -80.285502 | *A. carolinensis* |
| MK509140 | MIA720 | S78/H69 | South Miami, FL | 25.706275 | -80.285502 | *A. carolinensis* |
| MK509119 | MIA721 | S32/H33 | South Miami, FL | 25.706275 | -80.285502 | *A. carolinensis* |
| MK509134 | MIA722 | S62/H55 | South Miami, FL | 25.706499 | -80.2856 | *A. carolinensis* |
| MK509069 | MIA723 | S69 | South Miami, FL | 25.720751 | -80.279788 | *A. carolinensis* |
| MK509057 | MIA724 | S57 | South Miami, FL | 25.721547 | -80.279833 | *A. carolinensis* |
| MK509168 | MIA725 | S115 | South Miami, FL | 25.721547 | -80.279833 | *A. porcatus* |
| MK509045 | MIA729 | S35 | South Miami, FL | 25.718768 | -80.281233 | *A. carolinensis* |
| MK509067 | MIA731 | S67 | South Miami, FL | 25.717773 | -80.294104 | *A. carolinensis* |
| MK509073 | MIA732 | S78 | South Miami, FL | 25.717773 | -80.294104 | *A. carolinensis* |
| MK509055 | MIA733 | S50 | South Miami, FL | 25.722233 | -80.297597 | *A. carolinensis* |
| MK509071 | MIA734 | S75 | South Miami, FL | 25.722233 | -80.297597 | *A. carolinensis* |
| MK509056 | MIA735 | S56 | South Miami, FL | 25.724167 | -80.298412 | *A. carolinensis* |
| MK509044 | MIA740 | S31 | South Miami, FL | 25.724167 | -80.298412 | *A. carolinensis* |
| MK509144 | MIA743 | S114/H105 | South Miami, FL | 25.728767 | -80.300729 | *A. porcatus* |
| MK509046 | MIA744 | S42 | South Miami, FL | 25.736254 | -80.308689 | *A. carolinensis* |
| MK509059 | MIA747 | S54 | South Miami, FL | 25.736254 | -80.308689 | *A. carolinensis* |
| MK509048 | MIA748 | S48 | South Miami, FL | 25.740831 | -80.311349 | *A. carolinensis* |
| MK509084 | MIA749 | S115 | South Miami, FL | 25.740831 | -80.311349 | *A. porcatus* |
| MK509145 | MIA750 | S114/H105 | South Miami, FL | 25.740831 | -80.311349 | *A. porcatus* |

**Table S2**: Primer sequences and annealing temperatures (Tm) for the 18 microsatellite loci and partial mtDNA region of the NADH dehydrogenase subunit 2.

| Primer | Forward | Reverse | Reference | Tm |
| --- | --- | --- | --- | --- |
| f06 | GCCTTCCCTAAGCTATCCAAA | TGGCATTGAACCATCAGAA | This study | 60 |
| g01 | CAGATGGTTGACTCGATGTGTT | TTCAATAAAGTTGTGGCTGGTG | This study | 61 |
| Ac2 | TGTAAAACGACGGCCAGTGGCCACATAGTTGTGCCTCT | TTCACAATGTTTGTGGGTGT | This study | 60 |
| Ac5 | TGTAAAACGACGGCCAGTTGCTGGATTTCGTATCACAA | GTGGCCCATGAGTCACATCT | This study | 60 |
| Ac6 | TGTAAAACGACGGCCAGTTATTGTGATGTTGGGCAAGG | TGCTTCATGGTGATCTTGGA | This study | 60 |
| Acar1 | CCAAAAACCAAAAAGGCTGA | TGGACACACATACACCCACA | Wordley et al. 2011 | 57 |
| Acar4 | ACAGGGTACTGTGGACAGGG | AGGAGCGTGGAGCTACAAAA | Wordley et al. 2011 | 58 |
| Acar10 | GGATGTGTGTGTTTGTGTTGG | GGCTGTTGAGGGATTCTTGA | Wordley et al. 2011 | 57 |
| Acar11 | AGTTTCCCAAGAAAACCCGT | GGGTTGCTCGTTCTGGACTA | Wordley et al. 2011 | 59 |
| Acar14 | TATGTTGGGAGAAAGACGGG | CCTGAGCTACGTGACATGGA | Wordley et al. 2011 | 59 |
| Acar16 | CCAGAAAGCTTATTTCGGGTT | ATGTTGGATGAGCAAGGAGG | Wordley et al. 2011 | 58 |
| Acar19 | GAAAAGTAGTGGGGCATTGG | AGTTTCCCAAGAAAACCCGT | Wordley et al. 2011 | 57 |
| Acar22 | AACCACCTTTGTTCTGGTGC | AAGATGGCATTTCAGTGTTGC | Wordley et al. 2011 | 58 |
| Acar23 | TAATGGGGAGCAATTCAAGG | GAGCCCTATCTTTGGAAGGC | Wordley et al. 2011 | 58 |
| Acar28 | AACCCCATACATCGCCAATA | GAACTTGCATGAGGCTGTCA | Wordley et al. 2011 | 58 |
| Acar30 | CATCTCTTCAGGCTTTTGCC | CTGTCTCTTCCTCCACCTGC | Wordley et al. 2011 | 57 |
| Acar32 | ATCTGTGCTACACTGGCCCT | TCCCCACAGTCAAAAGAAGC | Wordley et al. 2011 | 58 |
| Acar43 | GAGAGGCCACCAGCATTTAC | GCATAAAGTGGGAATTGCTTC | Wordley et al. 2011 | 59 |
| ND2 | CCCACGATCTACAGAAGCAG | AGTAGGGAGGATGCGGCTAT | This study | 57 |

**Table S3**. Published sequences of mtDNA NADH dehydrogenase subunit 2 and haplotypes from two alignments. S = 343bp, H = 571bp.

| **Accession number** | **mtDNA Haplotype** | | **Reference** | **Species** |
| --- | --- | --- | --- | --- |
| AY654025 | S2 | H2 | Glor et al. 2004 | *A. porcatus* |
| AY654026 | S96 | H91 | Glor et al. 2004 | *A. porcatus* |
| AY654027 | S95 | H90 | Glor et al. 2004 | *A. porcatus* |
| AY654028 | S3 | H3 | Glor et al. 2004 | *A. porcatus* |
| AY654029 | S119 | H108 | Glor et al. 2004 | *A. porcatus* |
| AY654030 | S93 | H88 | Glor et al. 2004 | *A. porcatus* |
| AY654031 | S97 | H92 | Glor et al. 2004 | *A. porcatus* |
| AY654032 | S94 | H89 | Glor et al. 2004 | *A. porcatus* |
| AY654033 | S98 | H93 | Glor et al. 2004 | *A. porcatus* |
| AY654034 | S109 | H101 | Glor et al. 2004 | *A. porcatus* |
| AY654035 | S133 | H115 | Glor et al. 2004 | *A. porcatus* |
| AY654036 | S138 | H116 | Glor et al. 2004 | *A. porcatus* |
| AY654037 | S132 | H114 | Glor et al. 2004 | *A. porcatus* |
| AY654038 | S123 | H109 | Glor et al. 2004 | *A. porcatus* |
| AY654039 | S112 | H104 | Glor et al. 2004 | *A. porcatus* |
| AY654040 | S141 | H117 | Glor et al. 2004 | *A. porcatus* |
| AY654041 | S141 | H117 | Glor et al. 2004 | *A. porcatus* |
| AY654042 | S153 | H126 | Glor et al. 2004 | *A. porcatus* |
| AY654043 | S108 | H100 | Glor et al. 2004 | *A. porcatus* |
| AY654044 | S108 | H100 | Glor et al. 2004 | *A. porcatus* |
| AY654045 | S102 | H96 | Glor et al. 2004 | *A. porcatus* |
| AY654046 | S106 | H98 | Glor et al. 2004 | *A. porcatus* |
| AY654047 | S101 | H94 | Glor et al. 2004 | *A. porcatus* |
| AY654048 | S101 | H95 | Glor et al. 2004 | *A. porcatus* |
| AY654050 | S172 | H144 | Glor et al. 2004 | *A. porcatus* |
| AY654051 | S103 | H97 | Glor et al. 2004 | *A. porcatus* |
| AY654052 | S151 | H125 | Glor et al. 2004 | *A. porcatus* |
| AY654053 | S108 | H100 | Glor et al. 2004 | *A. porcatus* |
| AY654055 | S107 | H99 | Glor et al. 2004 | *A. porcatus* |
| AY654056 | S108 | H100 | Glor et al. 2004 | *A. porcatus* |
| AY654057 | S166 | H137 | Glor et al. 2004 | *A. porcatus* |
| AY654058 | S165 | H136 | Glor et al. 2004 | *A. porcatus* |
| AY654059 | S162 | H135 | Glor et al. 2004 | *A. porcatus* |
| AY654060 | S161 | H134 | Glor et al. 2004 | *A. porcatus* |
| AY654061 | S159 | H132 | Glor et al. 2004 | *A. porcatus* |
| AY654062 | S159 | H132 | Glor et al. 2004 | *A. porcatus* |
| AY654063 | S158 | H131 | Glor et al. 2004 | *A. porcatus* |
| AY654064 | S160 | H133 | Glor et al. 2004 | *A. porcatus* |
| AY654065 | S157 | H130 | Glor et al. 2004 | *A. porcatus* |
| AY654066 | S154 | H127 | Glor et al. 2004 | *A. porcatus* |
| AY654067 | S154 | H127 | Glor et al. 2004 | *A. porcatus* |
| AY654068 | S155 | H128 | Glor et al. 2004 | *A. porcatus* |
| AY654070 | S156 | H129 | Glor et al. 2004 | *A. porcatus* |
| AY654071 | S167 | H138 | Glor et al. 2004 | *A. porcatus* |
| AY654072 | S168 | H139 | Glor et al. 2004 | *A. porcatus* |
| AY654073 | S169 | H141 | Glor et al. 2004 | *A. porcatus* |
| AY654074 | S170 | H142 | Glor et al. 2004 | *A. porcatus* |
| AY654075 | S168 | H140 | Glor et al. 2004 | *A. porcatus* |
| AY654076 | S171 | H143 | Glor et al. 2004 | *A. porcatus* |
| AY654077 | S181 | H155 | Glor et al. 2004 | *A. porcatus* |
| AY654078 | S182 | H156 | Glor et al. 2004 | *A. porcatus* |
| AY654079 | S179 | H152 | Glor et al. 2004 | *A. porcatus* |
| AY654081 | S180 | H154 | Glor et al. 2004 | *A. porcatus* |
| AY654082 | S180 | H154 | Glor et al. 2004 | *A. porcatus* |
| AY654083 | S180 | H154 | Glor et al. 2004 | *A. porcatus* |
| AY654084 | S180 | H153 | Glor et al. 2004 | *A. porcatus* |
| AY654085 | S173 | H145 | Glor et al. 2004 | *A. porcatus* |
| AY654086 | S173 | H145 | Glor et al. 2004 | *A. porcatus* |
| AY654087 | S174 | H146 | Glor et al. 2004 | *A. porcatus* |
| AY654088 | S175 | H147 | Glor et al. 2004 | *A. porcatus* |
| AY654089 | S178 | H151 | Glor et al. 2004 | *A. porcatus* |
| AY654090 | S177 | H149 | Glor et al. 2004 | *A. porcatus* |
| AY654091 | S177 | H149 | Glor et al. 2004 | *A. porcatus* |
| AY654092 | S177 | H149 | Glor et al. 2004 | *A. porcatus* |
| AY654093 | S177 | H150 | Glor et al. 2004 | *A. porcatus* |
| AY654094 | S176 | H148 | Glor et al. 2004 | *A. porcatus* |
| AY902428 | S70 | H60 | Glor et al. 2005 | *A. carolinensis* |
| AY902429 | S74 | H63 | Glor et al. 2005 | *A. carolinensis* |
| AY902430 | S12 | H12 | Glor et al. 2005 | *A. carolinensis* |
| AY902431 | S10 | H10 | Glor et al. 2005 | *A. carolinensis* |
| AY902432 | S19 | H20 | Glor et al. 2005 | *A. carolinensis* |
| AY902433 | S16 | H21 | Glor et al. 2005 | *A. carolinensis* |
| AY902434 | S82 | H80 | Glor et al. 2005 | *A. carolinensis* |
| EU106323 | S11 | H11 | Kolbe et al. 2007 | *A. carolinensis* |
| EU106324 | S20 | H22 | Kolbe et al. 2007 | *A. carolinensis* |
| EU106325 | S16 | H18 | Kolbe et al. 2007 | *A. carolinensis* |
| EU106326 | S17 | H17 | Kolbe et al. 2007 | *A. carolinensis* |
| EU106327 | S18 | H19 | Kolbe et al. 2007 | *A. carolinensis* |
| EU106328 | S83 | H72 | Kolbe et al. 2007 | *A. carolinensis* |
| EU106329 | S89 | H83 | Kolbe et al. 2007 | *A. carolinensis* |
| EU106330 | S45 | H51 | Kolbe et al. 2007 | *A. carolinensis* |
| EU106331 | S76 | H65 | Kolbe et al. 2007 | *A. carolinensis* |
| EU106332 | S54 | H52 | Kolbe et al. 2007 | *A. carolinensis* |
| EU106333 | S30 | H32 | Kolbe et al. 2007 | *A. carolinensis* |
| EU106334 | S64 | H57 | Kolbe et al. 2007 | *A. carolinensis* |
| EU106335 | S78 | H69 | Kolbe et al. 2007 | *A. carolinensis* |
| EU106336 | S44 | H45 | Kolbe et al. 2007 | *A. carolinensis* |
| EU106337 | S50 | H53 | Kolbe et al. 2007 | *A. carolinensis* |
| EU106338 | S41 | H43 | Kolbe et al. 2007 | *A. carolinensis* |
| EU106339 | S55 | H54 | Kolbe et al. 2007 | *A. carolinensis* |
| EU106340 | S52 | H50 | Kolbe et al. 2007 | *A. carolinensis* |
| EU106341 | S43 | H44 | Kolbe et al. 2007 | *A. carolinensis* |
| EU106342 | S50 | H53 | Kolbe et al. 2007 | *A. carolinensis* |
| EU106343 | S110 | H102 | Kolbe et al. 2007 | *A. porcatus* |
| EU106344 | S118 | H106 | Kolbe et al. 2007 | *A. porcatus* |
| JX524289 | S33 | H34 | Campbell-Staton et al. 2012 | *A. carolinensis* |
| JX524291 | S24 | H26 | Campbell-Staton et al. 2012 | *A. carolinensis* |
| JX524292 | S25 | H27 | Campbell-Staton et al. 2012 | *A. carolinensis* |
| JX524293 | S22 | H24 | Campbell-Staton et al. 2012 | *A. carolinensis* |
| JX524294 | S26 | H28 | Campbell-Staton et al. 2012 | *A. carolinensis* |
| JX524295 | S23 | H25 | Campbell-Staton et al. 2012 | *A. carolinensis* |
| JX524296 | S73 | H62 | Campbell-Staton et al. 2012 | *A. carolinensis* |
| JX524297 | S68 | H59 | Campbell-Staton et al. 2012 | *A. carolinensis* |
| JX524298 | S62 | H55 | Campbell-Staton et al. 2012 | *A. carolinensis* |
| JX524299 | S40 | H42 | Campbell-Staton et al. 2012 | *A. carolinensis* |
| JX524300 | S80 | H68 | Campbell-Staton et al. 2012 | *A. carolinensis* |
| JX524301 | S33 | H34 | Campbell-Staton et al. 2012 | *A. carolinensis* |
| JX524302 | S50 | H53 | Campbell-Staton et al. 2012 | *A. carolinensis* |
| JX524303 | S36 | H37 | Campbell-Staton et al. 2012 | *A. carolinensis* |
| JX524304 | S62 | H55 | Campbell-Staton et al. 2012 | *A. carolinensis* |
| JX524310 | S6 | H6 | Campbell-Staton et al. 2012 | *A. carolinensis* |
| JX524311 | S4 | H4 | Campbell-Staton et al. 2012 | *A. carolinensis* |
| JX524312 | S9 | H9 | Campbell-Staton et al. 2012 | *A. carolinensis* |
| JX524313 | S7 | H7 | Campbell-Staton et al. 2012 | *A. carolinensis* |
| JX524314 | S5 | H5 | Campbell-Staton et al. 2012 | *A. carolinensis* |
| JX524315 | S16 | H16 | Campbell-Staton et al. 2012 | *A. carolinensis* |
| JX524316 | S82 | H71 | Campbell-Staton et al. 2012 | *A. carolinensis* |
| JX524317 | S82 | H71 | Campbell-Staton et al. 2012 | *A. carolinensis* |
| JX524318 | S14 | H14 | Campbell-Staton et al. 2012 | *A. carolinensis* |
| JX524319 | S13 | H13 | Campbell-Staton et al. 2012 | *A. carolinensis* |
| JX524320 | S15 | H15 | Campbell-Staton et al. 2012 | *A. carolinensis* |
| JX524321 | S88 | H82 | Campbell-Staton et al. 2012 | *A. carolinensis* |
| JX524322 | S82 | H71 | Campbell-Staton et al. 2012 | *A. carolinensis* |
| JX524323 | S82 | H71 | Campbell-Staton et al. 2012 | *A. carolinensis* |
| JX524324 | S82 | H71 | Campbell-Staton et al. 2012 | *A. carolinensis* |
| JX524325 | S8 | H8 | Campbell-Staton et al. 2012 | *A. carolinensis* |
| JX524326 | S82 | H71 | Campbell-Staton et al. 2012 | *A. carolinensis* |
| JX524327 | S82 | H71 | Campbell-Staton et al. 2012 | *A. carolinensis* |
| JX524328 | S82 | H71 | Campbell-Staton et al. 2012 | *A. carolinensis* |
| JX524329 | S84 | H74 | Campbell-Staton et al. 2012 | *A. carolinensis* |
| JX524330 | S87 | H79 | Campbell-Staton et al. 2012 | *A. carolinensis* |
| JX524331 | S86 | H78 | Campbell-Staton et al. 2012 | *A. carolinensis* |
| JX524332 | S82 | H76 | Campbell-Staton et al. 2012 | *A. carolinensis* |
| JX524368 | S82 | H75 | Campbell-Staton et al. 2012 | *A. carolinensis* |
| JX524369 | S85 | H77 | Campbell-Staton et al. 2012 | *A. carolinensis* |
| JX524370 | S82 | H81 | Campbell-Staton et al. 2012 | *A. carolinensis* |
| JX524371 | S82 | H73 | Campbell-Staton et al. 2012 | *A. carolinensis* |
| JX524372 | S82 | H73 | Campbell-Staton et al. 2012 | *A. carolinensis* |
| JX524373 | S91 | H85 | Campbell-Staton et al. 2012 | *A. carolinensis* |
| JX524374 | S91 | H86 | Campbell-Staton et al. 2012 | *A. carolinensis* |
| JX524375 | S21 | H23 | Campbell-Staton et al. 2012 | *A. carolinensis* |
| JX524376 | S90 | H84 | Campbell-Staton et al. 2012 | *A. carolinensis* |
| JX524377 | S92 | H87 | Campbell-Staton et al. 2012 | *A. carolinensis* |
| JX524408 | S72 | H61 | Campbell-Staton et al. 2012 | *A. carolinensis* |
| JX524410 | S74 | H64 | Campbell-Staton et al. 2012 | *A. carolinensis* |
| JX524412 | S77 | H66 | Campbell-Staton et al. 2012 | *A. carolinensis* |
| JX524414 | S27 | H29 | Campbell-Staton et al. 2012 | *A. carolinensis* |
| JX524419 | S82 | H71 | Campbell-Staton et al. 2012 | *A. carolinensis* |
| JX524420 | S81 | H70 | Campbell-Staton et al. 2012 | *A. carolinensis* |
| JX524421 | S82 | H71 | Campbell-Staton et al. 2012 | *A. carolinensis* |
| JX524422 | S82 | H71 | Campbell-Staton et al. 2012 | *A. carolinensis* |

**Table S4:** Residual matrix of tree-based population models. Positive values indicate greater genetic variation in the true population than explained by the model. Lower positive residuals show improvement of the model after including migration.

| **No Migration** | | | |
| --- | --- | --- | --- |
|  | **SFL** | **MIA** | **WCU** |
| **SFL** | 0.29 |  |  |
| **MIA** | **2.05** | -4.10 |  |
| **WCU** | -2.34 | **2.05** | 0.29 |
| **Migration** | | | |
|  | **SFL** | **MIA** | **WCU** |
| **SFL** | -0.32 |  |  |
| **MIA** | -0.07 | -0.28 |  |
| **WCU** | **0.39** | **0.35** | -0.74 |

**Table S5.** Deviation of summary statistics between the observed data and simulated data from the posterior predictive distributions in the ABC analyses. A = mean number of alleles, H = mean gene diversity, FST = pairwise F_ST_ – value, $\lambda$ = maximum likelihood coefficient of admixture.

| **Summary** | **Observed** | **Proportion** | **Significance** |
| --- | --- | --- | --- |
| **Statistics** | **Value** | **(simulated<observed)** |  |
| A_SFL_ | 10.22 | 0.63 |  |
| A_MIA_ | 13.17 | 0.06 |  |
| A_WCU_ | 12.61 | 0.31 |  |
| H_SFL_ | 0.80 | 0.42 |  |
| H_MIA_ | 0.81 | 0.03 | * |
| H_WCU_ | 0.86 | 0.54 |  |
| F_ST-SFL_ _x MIA_ | 0.09 | 0.11 |  |
| F_ST-SFL_ _x WCU_ | 0.08 | 0.00 | *** |
| F_ST-MIA_ _x WCU_ | 0.07 | 0.81 |  |
| $\bar{\lambda}$ | 0.31 | 0.87 |  |

**Table S6**. Bias and precision of parameter estimates of the ABC analysis. Bias = the average relative bias, MMedAD = relative median absolute deviation, RMAE = relative median of the absolute error.

| **Parameter** | **Bias** | **MMedAD** | **RMAE** |
| --- | --- | --- | --- |
| N_SFL_ | 0.11 | 0.40 | 0.21 |
| N_MIA_ | 0.25 | 0.59 | 0.30 |
| N_WCU_ | 0.00 | 0.23 | 0.12 |
| T_A_ | 0.29 | 0.95 | 0.42 |
| R_A_ | -0.07 | 0.30 | 0.17 |
| T_MRCA_ | -0.02 | 0.31 | 0.20 |
| µmic_1 | -0.07 | 0.26 | 0.16 |
| pmic_1 | 0.06 | 0.40 | 0.24 |
| snimic_1 | 111.89 | 225.67 | 21.72 |

**Figure S1**. Maximum likelihood phylogeny of mtDNA haplotypes of the 343bp alignment. The phylogeny includes all 280 individuals and were collapsed into 181 unique haplotypes. Bootstrap values are shown above branches for values >95.

**Figure S2**. Maximum likelihood phylogeny of mtDNA haplotypes of the 571bp alignment. The phylogeny includes 200 individuals and were collapsed into 156 unique haplotypes. Bootstrap values are shown above branches for values >95.

**Figure S3**. Allele frequency distributions for 18 microsatellite markers. *Anolis carolinensis* from SFL is shown in yellow, *A. porcatus* from WCU is shown in green and the hybrid population from MIA is shown in magenta.

**Figure S4**. Model comparison for a sequentially increasing number of clusters (K). left: delta K for STRUCTURE models with varying number of clusters. Large delta K indicates that K = 3 clusters is the best fitting model. Right: BIC values for cluster models generated by DAPC. The best fitting model indicated by the smallest BIC and has three clusters.


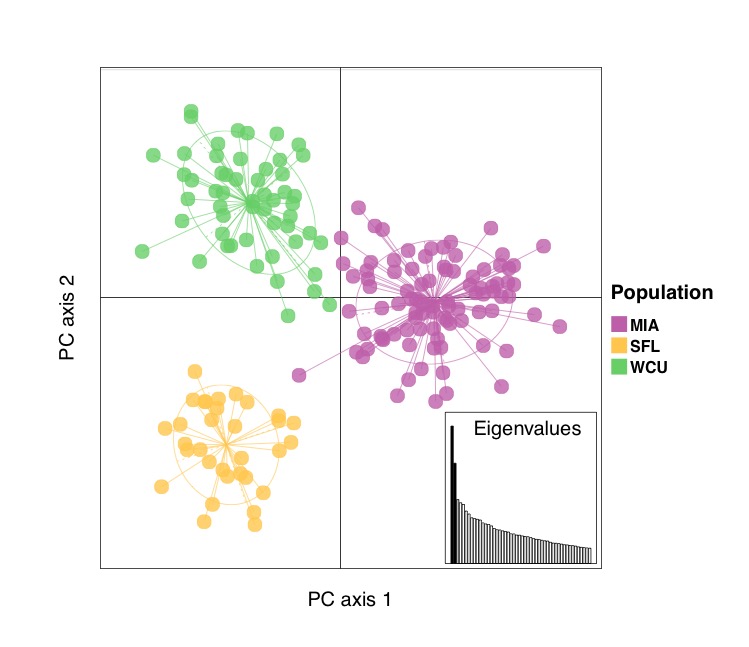


**Figure S5**. Principal component analysis of microsatellite genotypes from western Cuba (WCU), southern Florida (SFL) and South Miami (MIA) shows genetic variation within and between populations. The three sampling locations form separate genetic clusters.

**Figure S6**. STRUCTURE analysis of population pairs. Populations cluster according to the sampling location and no further substructure was detected.

**Figure S7**. Log likelihood L(K) of STRUCTURE models with increasing number of clusters K for the WCU sampling locations. L(K) decreases with increasing number of clusters suggesting absence of population structure within WCU.

**Figure S8.** Simple tree model used to test for historic gene flow. The simple tree model accounted for 80% of the variance in the data.
